# Long non-coding RNA CCL14-AS suppresses invasiveness and lymph node metastasis of colorectal cancer cells by regulating MEP1A

**DOI:** 10.1186/s12935-023-02866-1

**Published:** 2023-02-15

**Authors:** Mingzhou Li, Chengmei Huang, Yuanyuan Wu, Lina Zhu, Yaxin Zhang, Yi Zhou, Huali Li, Zhihao Liu, Xinyan Pan, Xin Wang, Junfeng Qiu, Fengtian Li, Wenting Liao

**Affiliations:** 1 State Key Laboratory of Oncology in South China, Collaborative Innovation Center for Cancer Medicine, Sun Yat-sen University Cancer Center, Guangzhou, China; 2 Department of Pathology, Nanfang Hospital and School of Basic Medical Sciences, Southern Medical University, Guangzhou, 510515 China; 3 Department of Biomedical Sciences, City University of Hong Kong, Hong Kong, China

**Keywords:** Long noncoding RNA, AC244100.2, CCL14-AS, Colorectal cancer, Metastasis, MEP1A

## Abstract

**Background:**

Long non-coding RNAs (lncRNAs) play important roles in the biology of colorectal cancer (CRC). There are several lncRNAs associated with invasion and metastasis have been characterized in CRC. However, studies focusing on the precise molecular mechanisms by which lncRNAs function in lymph node (LN) metastasis in CRC are still limited.

**Methods:**

In this study, by analyzing TCGA dataset, we identified that AC244100.2 (termed CCL14-AS), a novel lncRNA enriched in the cytoplasm, was negatively correlated with LN metastasis and unfavorable prognosis of CRC. In situ hybridization was used to examine CCL14-AS expression in clinical CRC tissues. Various functional experiments including migration assay and wound-healing assay were used to investigate the effects of CCL14-AS on CRC cells migration. The nude mice popliteal lymph node metastasis model assay further confirmed the effects of CCL14-AS in vivo.

**Results:**

CCL14-AS expression was significantly downregulated in CRC tissues compared to adjacent normal tissues. In addition, low CCL14-AS expression was correlated with advanced T classification, LN metastasis, distant metastasis, and shorter disease-free survival of CRC patients. Functionally, CCL14-AS overexpression inhibited the invasiveness of CRC cells in vitro and LN metastasis in nude mice. On the contrary, knockdown of CCL14-AS promoted the invasiveness and LN metastasis abilities of CRC cells. Mechanistically, CCL14-AS downregulated the expression of MEP1A via interacting with MEP1A mRNA and reduced its stability. Overexpression of MEP1A rescued the invasiveness and LN metastasis abilities in CCL14-AS-overexpressing CRC cells. Moreover, the expression levels of CCL14-AS was negatively correlated with that of MEP1A in CRC tissues.

**Conclusions:**

We identified a novel lncRNA, CCL14-AS, as a potential tumor suppressor in CRC. Our findings supported a model in which the CCL14-AS/MEP1A axis serves as critical regulator in CRC progression, suggesting a novel biomarker and therapeutic target in advanced CRC.

**Supplementary Information:**

The online version contains supplementary material available at 10.1186/s12935-023-02866-1.

## Background

Colorectal cancer (CRC), the third most diagnosed cancer and the second leading cause of cancer-associated mortality worldwide, accounts for 10% of all new cancer cases diagnosis and 9.4% of all cancer deaths in 2020 [1]. Approximately 20% CRC patients are with stage IV disease which is present with metastasis when diagnosed [2] and over half of patients with advanced CRC die from recurrence and metastasis. Early diagnosis and treatment of patients with invasive CRC tumor in early stages is vital to reduce the mortality of CRC patients [3]. However, the potential risk of lymph node (LN) metastasis is still an important clinical consideration, especially in patients with early-stage CRC [4]. LN metastasis occurs in 6.8–19.6% early-stage CRC patients [5, 6]. In CRC patients without distant metastasis, LN metastasis is a significant factor for predicting survival. In addition, regional LN metastasis is an essential step in tumor cell dissemination [7]. Therefore, it is of great value to identify novel biomarkers and potential targets for LN metastasis in CRC.

Long non-coding RNAs (lncRNAs) are defined as transcripts longer than 200 nucleotides that have no or limited protein-coding capacity [8, 9]. With the great progress in understanding the role of lncRNAs, increasing evidence have shown that lncRNAs are key regulators of cancer pathways and as biomarkers of diseases [10]. LncRNAs may contribute to carcinogenesis and metastasis in cancer by regulating protein-coding genes expression at both the transcriptional and post-transcriptional levels [10–12]. For examples, lncRNA H19, HOTAIR, and MALAT1 were widely reported to be involved in carcinogenesis and metastasis of human cancers[13–21]. LncRNA H19 has been reported to play an oncogenic role by promoting cell proliferation, motility, invasion, and metastasis, in many types of cancers [13–17]. LncRNA HOTAIR is upregulated in multiple cancers and is a powerful predictor of tumor aggressiveness and metastasis [3, 18–20]. HOTAIR promotes invasion and metastasis in a manner dependent on Polycomb repressive complex 2 (PRC2) [18]. MALAT1 suppresses breast cancer metastasis by binding and inactivating the pro-metastatic transcription factor TEAD [22]. LncRNAs, such as HOXD-AS1, LINC00460, ZEB1-AS1, MIR17HG, have been demonstrated to have essential roles in regulating the proliferation, migration, invasion, and metastasis of CRC cells [23–26]. However, studies focusing on the precise molecular mechanisms by which lncRNAs function in LN metastatic CRC are still limited and would be critical for exploring novel biomarkers and therapeutic targets.

In this context, by analyzing TCGA dataset, we identified a novel lncRNA AC244100.2 that was negatively correlated with LN metastasis and unfavorable prognosis of CRC. AC244100.2 is an antisense lncRNA of C–C motif chemokine ligand 14 (CCL14) and overlaps with the 5ʹ UTR and first coding exon of the CCL14 mRNA. Thus, we termed it as CCL14-AS. CCL14-AS is located at human chromosome 17q12 and is transcribed into a 3878 bp transcript. The function and underlying mechanism of CCL14-AS in cancer progression are unclear. In the current study, we sought to figure out the expression pattern and biological role of CCL14-AS in the progression of CRC. We found that CCL14-AS was enriched in the cytoplasm of CRC cells. Overexpression of CCL14-AS significantly weakened migration in vitro and LN metastasis ability of CRC cells in nude mice. Moreover, CCL14-AS downregulated the expression of MEP1A via interacting with MEP1A mRNA and reduced its stability. Overexpression of MEP1A rescued the migration and LN metastasis abilities in CCL14-AS-overexpressing CRC cells. Our data demonstrated that CCL14-AS acted as a potential tumor suppressor in CRC. CCL14-AS/MEP1A axis may serve as promising regulator in CRC progression, suggesting a novel biomarker and therapeutic target in advanced CRC.

## Materials and methods

### Patients and specimens

The tissue microarrays containing a total of 96 CRC samples were collected and diagnosed in the Department of Pathology, Nanfang Hospital Southern Medical University. None of these patients received radiotherapy and chemotherapy before surgery. The clinical features and pathological information were obtained retrospectively from the medical records of these patients. In addition, eight pairs of fresh tumor tissues and non-cancerous adjacent tissues from patients with CRC were obtained from the operating room of Nanfang Hospital. The fresh biopsies were stored in liquid nitrogen before use. Written-informed consent was obtained from all participants prior to sample collection and the use of clinical materials for research purposes were approved by the Southern Medical University Institutional Board (Guangzhou, China).

### Cell lines and cell culture

The human CRC cell lines and human immortalized colon epithelial cell line NCM460 cells were purchased from the American Type Culture Collection and stored in the Department of Pathology, Nanfang Hospital Southern Medical University. RKO, SW837, DLD1, COLO205 and LoVo cells were cultured in RPMI 1640 medium (Gibco) with 10% fetal bovine serum (FBS) (Gibco), 100 U/mL penicillin and 100 μg/ml streptomycin. SW480 and SW620 cells were cultured in Leibovitz's L-15 medium (Gibco) with 10% FBS (Gibco), 100 U/ml penicillin and 100 μg/ml streptomycin. NCM460 and HCT116 cells were cultured in McCoy’s 5A medium (Gibco) supplemented with 10% FBS (Gibco),100 U/mL penicillin and 100 μg/mL streptomycin. Cells were cultured at a 37 °C humidified incubator with 5% CO2 and periodically tested mycoplasma negative.

### Lentivirus production and construction of stable cell lines

To evaluate the expression of CCL14-AS, CCL14-AS constructs were generated by cloning full-length human CCL14-AS cDNA into Ubi-MCS-SV40-puromycin vector. For deletion of CCL14-AS, two short hairpin RNA (shRNA) (sequences: CCL14-AS shRNA#1: 5ʹ-GCTCTGGAATTAGCCATTT-3ʹ; CCL14-AS shRNA#2: 5ʹ-GCTCCAACATAATGACAAA-3ʹ) were cloned into hU6-MCS-Ubiquitin-EGFP-IRES-puromycin. The cDNA encoding the ORF of MEP1A was amplified by PCR and inserted into pLVX-IRES-Neo lentivirus vector. The following primers were used for MEP1A cloning: Forward primer, 5ʹ-CCGGTGAATTCCTCGAGCCACCATGGCTTGGATTAGATCCACTTG-3ʹ; Reverse primer: 5ʹ-GGCGGGATCCTACTTGTCATCGTCGTCCTTGTAATCCTTCCTTGGCCTTTGGGAAAGG-3ʹ. Lentivirus were generated by co-transfecting 293 T cells with CCL14-AS or MEP1A-overexpressing plasmids and two packing vectors (psPAX and pMD2G) using Lipofectamine 2000 (Invitrogen). The supernatants of 293 T cultured medium after 48 h, filtering through 0.45-mm filters (Millipore, Temecula, CA, USA) and concentrated for further using. The expression of CCL14-AS in stable cell lines was detected by qRT-PCR. The expression of MEP1A was detected by qRT-PCR and western blot.

### RNA isolation, reverse transcription (RT) and real-time PCR

RNA isolation was carried out as previously described [27]. qRT-PCR was performed at least three times in triplicate using SYBR GREEN MIXTURE (Vazyme, Q311-03) and CFX96 Touch Real-Time PCR detection system. The data were normalized to the geometric mean of the housekeeping gene GAPDH and calculated using the 2−^ΔΔCT^ method. The following primer sequences were used for amplification: CCL14-AS Forward primer, 5ʹ- AGCATGAGTGGTCTTTAATTCAAA -3ʹ; CCL14-AS reverse, 5ʹ-GGCTGCCATTCCCTTCTT-3ʹ; MEP1A Forwardprimer, 5ʹ-TGACAGCACAGGCAATGTTCGC-3ʹ; MEP1A reverse, 5ʹ-GTCGCCTTTTGTGCCCTGGAAA-3ʹ; GAPDH Forward primer, 5ʹ-AACGGGAAGCTTGTCATCAA-3ʹ; GAPDH reverse, 5ʹ-TGGACTCCACGACGTACTCA-3ʹ.

### Western blot

Western blot was performed as described previously [28] using anti-MEP1A (Bioss, bs-6056R) and anti-GFP (Abcam, ab290). Anti-GAPDH and anti-α-tubulin antibody (Sigma, #9026) were used as the internal control.

### Immunohistochemistry

Immunohistochemical (IHC) staining was performed as previously described using a specific MEP1A antibody (Bioss, bs-6056R) [28]. After reviewed and scored independently by two observers, MEP1A protein expression levels were quantified based on the intensity of staining (0–3). The staining intensity was graded according to the following criteria: 0 (no staining); 1 (weak staining = light yellow), 2 (moderate staining = yellow brown), and 3 (strong staining = brown). Determine the best cut-off value: intensity of staining ≥ 2 is used to define tumors with high MEP1A expression, and index ≤ 1 is used to define tumors with low MEP1A expression.

### RNA fluorescence in situ hybridization (FISH)

The probe of CCL14-AS was designed and synthesized by GenePharma (Shanghai, China). The sequence is: 5′-CGTTCTTTGGATCGTGAGGATGATTTGTCAGCTCAGAGGAACAACTAGGGAAAGGCTTCTATCAAATACCAACTGTCAAAAGCTTAGGGGAAAAGTACAGGAACTTCC-3′. The hybridization and fluorescence detection were performed using FISH Kits from GenePharma (F22201/100, Shanghai, China) according to the manufacturer’s protocol. Finally, the signal was observed and photographed under a confocal microscope or fluorescent microscopy (Olympus, Japan). ImageJ software was used to measure the fluorescence intensity and distribution. The average of intensity and standard deviation were calculated and divided into low, medium, and high groups.

### Cellular proliferation, colony formation assays

For cell proliferation assays, a total of 3000 cells/well were plated into 96-well plates and cultured for 24 h, then added 20 µl of MTT in each well and incubated at 37 °C for 4 h. After removing the supernatant, in each well, we added 150 µl of DMSO and incubated at 37 °C for 10 min. Finally, we examined the absorbance at 570 nm with a microplate reader (800TS, Biotek, USA). For the clone-formation experiment, 1000 cells/well were seeded into 6-well plates and cultured for 2 weeks. Clones were fixed in 4% paraformaldehyde (PFA) and stained with 0.5% crystal violet. colonies were counted manually and each single colony consists of at least 50 cells.

### Cell migration and wound healing assays

Cell migration assays were performed using transwell. Cells (1 × 10^5^) in culture medium containing 1% FBS were added to the top chamber of 24-well transwell chambers plates (8.0 μm, Corning, NY, USA.) and culture medium with 10% FBS was added in the lower chamber as a chemoattractant.

After incubation for 24 h, the underside cells of the membrane were fixed, stained with 0.1% Crystal Violet, and then the percentages of migrated cells were counted (10 random 200 × fields per well). Three independent experiments were performed, and the data were presented as the mean ± SEM. For wound healing assays, a total of 1 × 10^6^ cells/well were plated into 6-well plates and cultured to about 80% confluence. After serum starvation for 24 h, scratches were performed in the middle slides using 10 μl pipette tip for each well. The closure of the gap distance was observed and photographed at 0 h, 12 h, 24 h, 36 h and 48 h under an inverted light microscope and quantitatively evaluated using ImageJ. Each experiment was repeated at least three times.

### Isolation of cytoplasmic and nuclear

The nuclear and cytosolic fractions of LoVo cells were isolated with Nuclear/Cytosol Extraction Kit (Bestbio, China) following the manufacturer’s protocol.

### RNA sequencing analysis

Total RNA was isolated from LoVo stable cells expressing vector or CCL14-AS. The sequencing libraries were generated using NEBNext^®^ UltraTM RNA Library Prep Kit for Illumina^®^ (NEB, USA) following manufacturer’s recommendations and index codes were added to attribute sequences to each sample. The samples were paired end sequenced with a read length of 150 bp on an Illumina Novaseq platform. The process of sequencing was controlled by Illumina Data collection software. The library construction and sequencing were performed at GenePharma (Shanghai, China). After pre-treatment of the raw reads (filtering and QC) and alignment to the genome, the expression level of genes was determined based on the value of FPKM (Fragments Per Kilobase of transcript per Million mapped reads). Differential expression analysis of two groups was performed using the DESeq2 R package. Genes with an adjusted P-value < 0.05 found by DESeq2 were assigned as differentially expressed. Gene Ontology (GO) enrichment analysis of differentially expressed genes was implemented by using the DAVID GO database to search for enriched pathways.

### RNA immunoprecipitation (RIP)

pMS2-GFP (Addgene) was transfected into stably overexpress CCL14-AS cells. After 48 h, cells were used to perform RIP experiments using a GFP antibody (5 µg per reaction, Abcam, ab290). LoVo cells were lysed in lysis Buffer with RNase inhibitor (Beyotime, 1:1000), DMSF (1:100) and Protease inhibitor (Beyotime,1:100). The protein A/G sepharose beads were washed by lysis Buffer, and the cell extracts were incubated with these beads bound to GFP antibody or control IgG at 4 °C for 2 h. The beads were washed and divided two parts. One part was incubated with proteinase K to remove protein and performed RNA isolation. Other part was used for western blot. Finally, qRT-PCR analysis was performed on the purified RNA, and western blot analysis for Anti-IgG and anti-GFP are performed to detect the quality of the experiment.

### RNA-stability analysis

Cells were treated with the transcriptional inhibitor actinomycin D (Sigma, USA) for 0 min, 20 min, 40 min, 1 h, 2 h, 4 h, 6 h, 8 h, 10 h and 12 h, respectively. Cells were harvested at different time points, and RNA was isolated. Then, qRT-PCR analysis was used to detect the level of MEAP1 mRNA as mentioned above.

### Mouse popliteal lymphatic metastasis model

The female BALB/c nude mice (4 to 6-week-old) were obtained from the Animal Center of Southern Medical University, Guangzhou, China. All animal experiments were approved by the Animal Care and Use Committee at Southern Medical University. Groups of LoVo-Vector, LoVo-CCL14-AS, LoVo-CCL14-AS-MEP1A, HCT116-Scramble and HCT116-shCCL14-AS cells (5 × 10^6^ per mouse) were injected into the footpads of mice correspondingly. After 8 weeks, the mice were sacrificed, and their footpad tumors and inguinal lymph nodes were detached. These tissues were fixed with formaldehyde and paraffin-embedded, and then 5-mm sections were cut and stained with haematoxylin and eosin (H&E).

### Statistical analysis

All statistical analyses were conducted by using SPSS version 20.0 (SPSS Inc, IL, USA) and GraphPad Prism Version 8.0 (GraphPadSoftware, Inc.USA). The differential expression between the two groups was calculated using Student’s t test. One-way ANOVA test was used to compare the differences of data from multiple groups, and LSD-T was used for multiple comparisons. The relationship between pathological parameters was compared using two independent samples nonparametric test and a chi-square test. The Kaplan–Meier method was used for analyzing the survival curves of CRC patients in low- and high-CCL14-AS or MEP1A expression groups and the log-rank test log-rank test was used to compare differences. A p value of < 0.05 was considered significant. (* *p* < 0.05, ** *p* < 0.01, *** *p* < 0.001).

## Results

### CCL14-AS is downregulated in CRC and associated with favorable prognosis

**Fig. 1:**
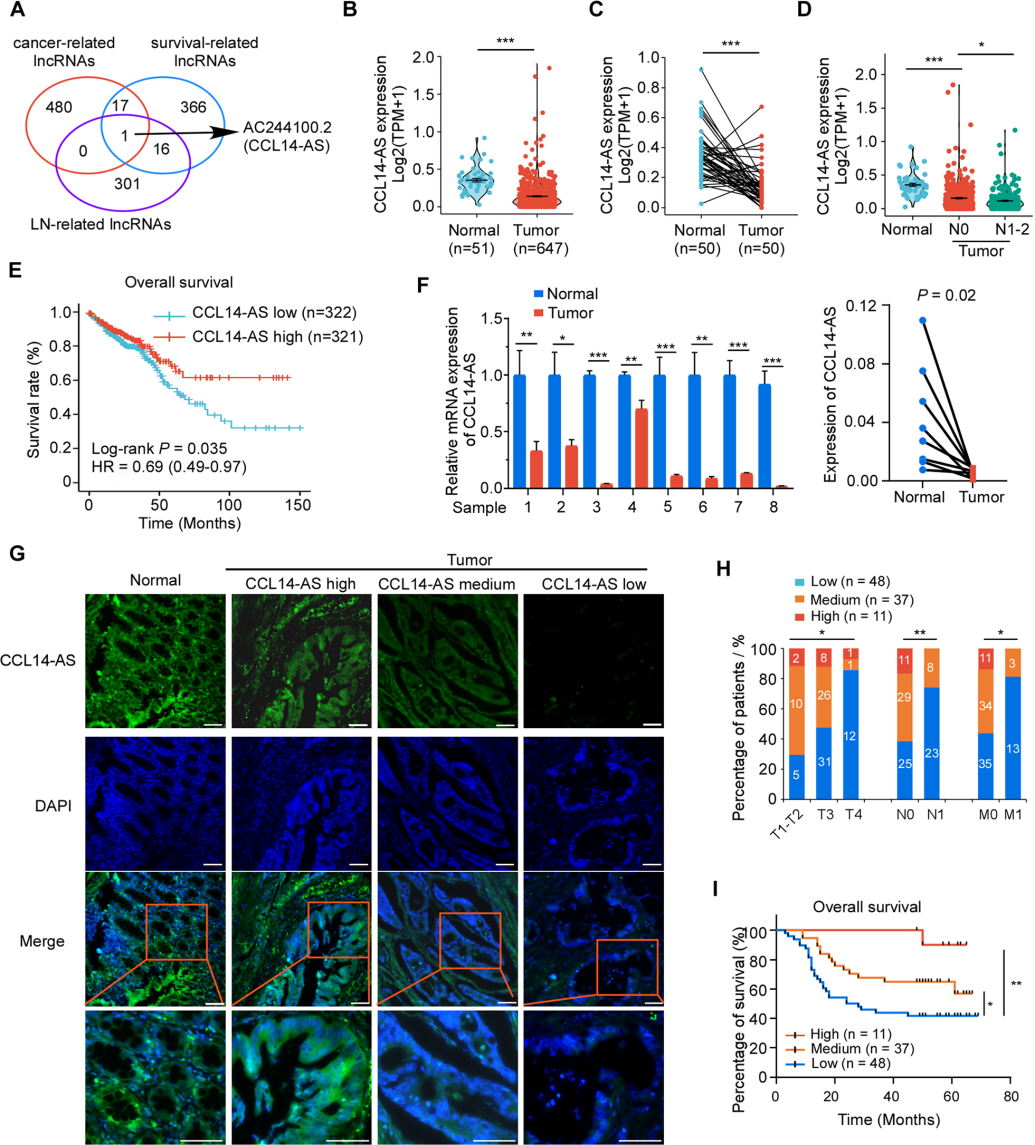
CCL14-AS is downregulated in CRC and associated with favorable prognosis.** A** The cancer-related, metastasis-related and survival-related lncRNAs were selected from the TCGA datasets. **B** Analysis of CCL14-AS expression in CRC compared with normal tissues in TCGA-COADREAD. **C** CCL14-AS expressions in paired CRC cancer tissues and adjacent normal tissues in TCGA-COADREAD were compared. **D** CCL14-AS expression in normal, N0 Stage CRC samples and N1-2 Stage CRC samples in TCGA-COADREAD was also compared. **E** Kaplan–Meier analysis of overall survival in all patients with CRC according to CCL14-AS expression in TCGA datasets (log-rank test* P* = 0.035).** F** qRT-PCR was performed to determine relative CCL14-AS RNA level in paired CRC cancer tissues and adjacent normal tissues. **G**–**I** FISH assay was conducted to determine the expression of CCL14-AS in 96 paraffin-embedded CRC tissues. **G** Representative FISH images are shown. CCL14-AS‑stained are green, DAPI‑stained nuclei are blue. Scale bar: 100 μm. **H** Graphical illustration of statistical CCL14-AS distribution in CRC patients. **I** Kaplan–Meier analysis of overall survival rate according to high, medium or low CCL14-AS expression. * p < 0.05, ** p < 0.01, *** *p* < 0.001

To identify novel biomarkers which can effectively predict CRC metastasis, The Cancer Genome Atlas (TCGA) datasets with expression profiles of lncRNAs in exoRBase CRC data were analyzed. The relative expressions of lncRNAs were analyzed by comparing cancer samples with normal samples, and metastatic (M1) samples with non-metastatic (M0) samples. Fold change ≥ 0.5 and p< 0.01 were considered differently expressed genes. LncRNAs differentially expressed in CRC tissues compared with normal tissues (Additional file 4: Table S1, cancer-related lncRNAs) and differently expressed in LN negative samples compared with LN positive samples (Additional file 5: Table S2, LN-related lncRNAs) were identified. Univariate Cox proportional-hazards regression model were performed to analyze the lncRNA that were associated with patient survival (Additional file 6: Table S3, survival-related lncRNAs). Integrating these three groups of lncRNAs triangulated on a single lncRNA, AC244100.2 (Fig. 1A). As AC244100.2 is an antisense lncRNA of C–C motif chemokine ligand 14 (CCL14) and overlaps with the 5ʹ UTR and first coding exon of the CCL14 mRNA, we termed it as CCL14-AS (Additional file 1: Fig S1A). The coding potential of CCL14-AS was calculated using the National Center for Biotechnology Information (NCBI) ORF finder, showing that CCL14-AS fails to translate protein of more than 85 amino acids (Additional file 7: Table S4). Analyses using the Coding Potential Assessment Tool (CPAT) showed that protein coding probability of CCL14-AS is 2.69% (Additional file 1: Figure S1B). This result suggested that CCL14-AS is a noncoding RNA. The secondary structure of CCL14-AS was predicted using online software RNAfold (Additional file 1: Fig S1C).

To evaluate the potential role CCL14-AS in CRC, the expression pattern of CCL14-AS and its clinical relevance were analyzed in TCGA COADREAD dataset and clinical CRC samples. In the TCGA COADREAD dataset, CCL14-AS was significantly downregulated in CRC tissues as compared with normal tissues (Fig. 1B, C). In addition, low CCL14-AS expression level is significantly correlated with lymph node metastasis (Fig. 1D) and poor Overall survival rate (OS) in TCGA dataset (Fig. 1E) in TCGA CRC patients. Next, the expression of CCL14-AS was validated in 8 paired clinical CRC samples by qRT-PCR, showing that CCL14-AS was dramatically downregulated in CRC tissues relative to adjacent non-cancerous tissues (Fig. 1F). CCL14-AS expression was further examined via in situ hybridization (ISH) in an expanded cohort of 96 paraffin-embedded CRC samples. The results showed that CCL14-AS is highly expressed in normal intestine tissues. However, it was dramatically downregulated in CRC tissues (Fig. 1G). Moreover, low CCL14-AS expression was significantly correlated with advanced T classification, lymph node metastasis, and distant metastasis (Fig. 1H). Furthermore, Kaplan–Meier analysis showed that low CCL14-AS expression was significantly associated with poor overall survival rate (OS) in this cohort of CRC samples (Fig. 1I). These results showed that CCL14-AS is a novel lncRNA associated with CRC progression.

### CCL14-AS suppresses invasiveness and LN metastasis of CRC cells

**Fig. 2:**
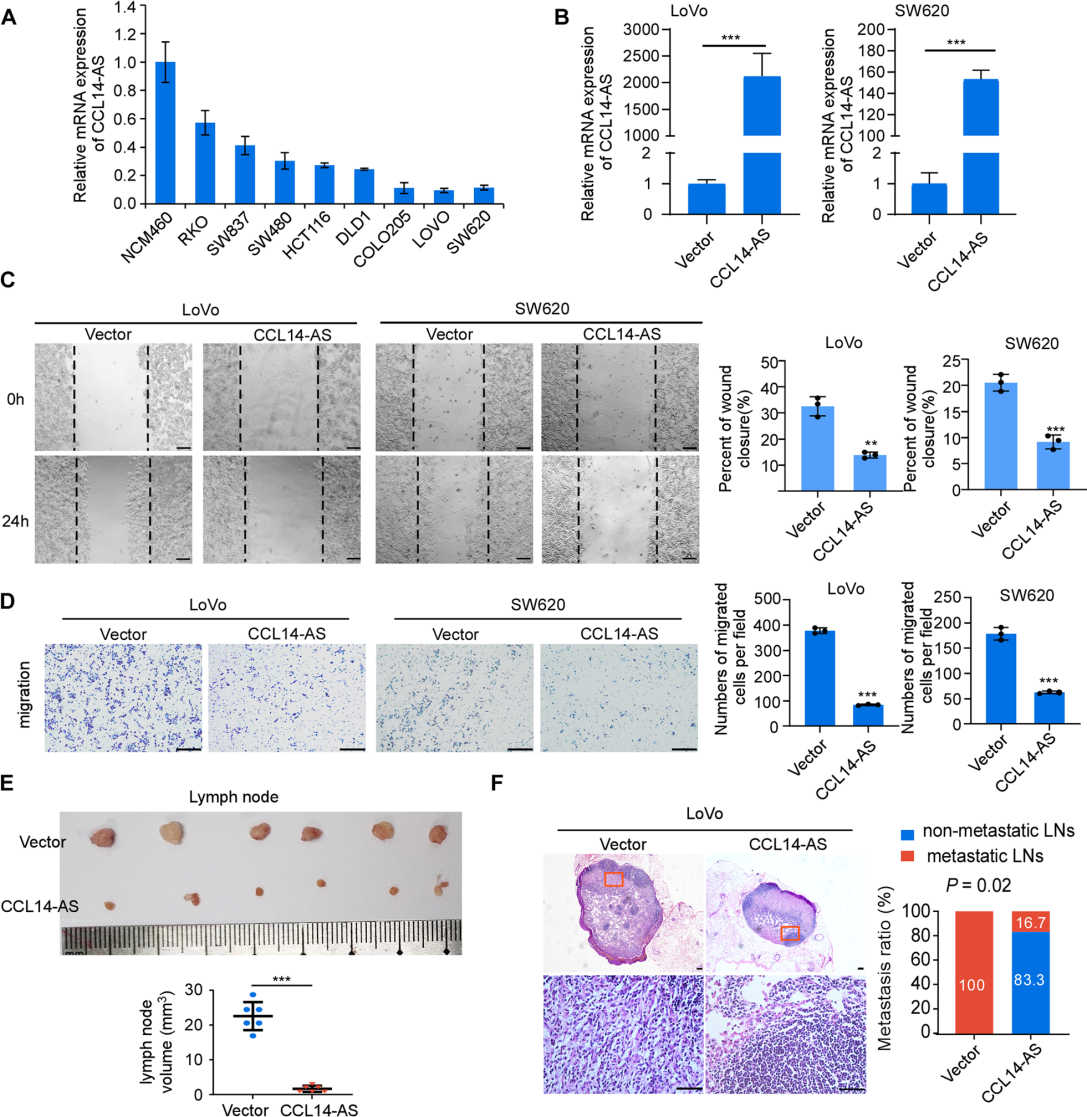
Overexpression of CCL14-AS suppresses invasiveness and lymph node metastasis of CRC cells. **A** A basal level of CCL14-AS was determined using qRT-PCR assay in the non-malignant human colon epithelial cell line NCM460 and several CRC cell lines (including SW480, SW837, SW620, HCT116, DLD-1, LoVo, COLO205 and RKO). **B** qRT-PCR analysis was performed to detect the expression levels of CCL14-AS in LoVo and SW620 cells with or without CCL14-AS stable overexpression. **C** Wound healing assay showing cell migration of LoVo(left) and SW620(right) cells with or without CCL14-AS overexpression. Scale bar: 100 μm. **D** Migration and invasion of LoVo (left) and SW620 cells (right) with or without CCL14-AS overexpression were evaluated by using transwell assays. Scale bar: 500 μm. **E** Popliteal lymph nodes volume and size were significantly decreased in the CCL14-AS overexpression group compared with the control group. **F** Representative images of the lymph node stained with H&E(left) in the CCL14-AS overexpression group. Scale bar: 100 μm. Statistical results of the metastatic ratios of lymph nodes in the Vector control and CCL14-AS-Overexpresed group (right). * *p* < 0.05, ** p < 0.01, *** *p* < 0.001

The endogenous expression of CCL14-AS in CRC cell lines were examined by qRT-PCR. The results showed that the CCL14-AS expression level was much higher in the human immortalized colon epithelial cell NCM460 than in CRC cells (Fig. 2A). To investigate the biological functions of CCL14-AS on the progression of CRC, CCL14-AS was stably overexpressed in LoVo and SW620 cell lines, which showed low endogenous expression of CCL14-AS (Fig. 2B). MTT assays and colony formation assays revealed that overexpression of CCL14-AS does not affect the proliferation of CRC cells (Additional file 2: Figure S2A–S2B). However, wound healing assays and migration assays showed that overexpression of CCL14-AS dramatically decreased the migration and invasion of CRC cells (Fig. 2C, D). As the CCL14-AS expression levels are closely correlated with LN metastasis both in TCGA COAD datasets and in clinical CRC samples, we examined the role of CCL14-AS in LN metastasis potential using the nude mice popliteal lymph node metastasis model. Expectedly, overexpression of CCL14-AS significantly inhibited LN metastasis, as determined the volumes and numbers of the metastatic lymph nodes (Fig. 2E, F).

**Fig. 3:**
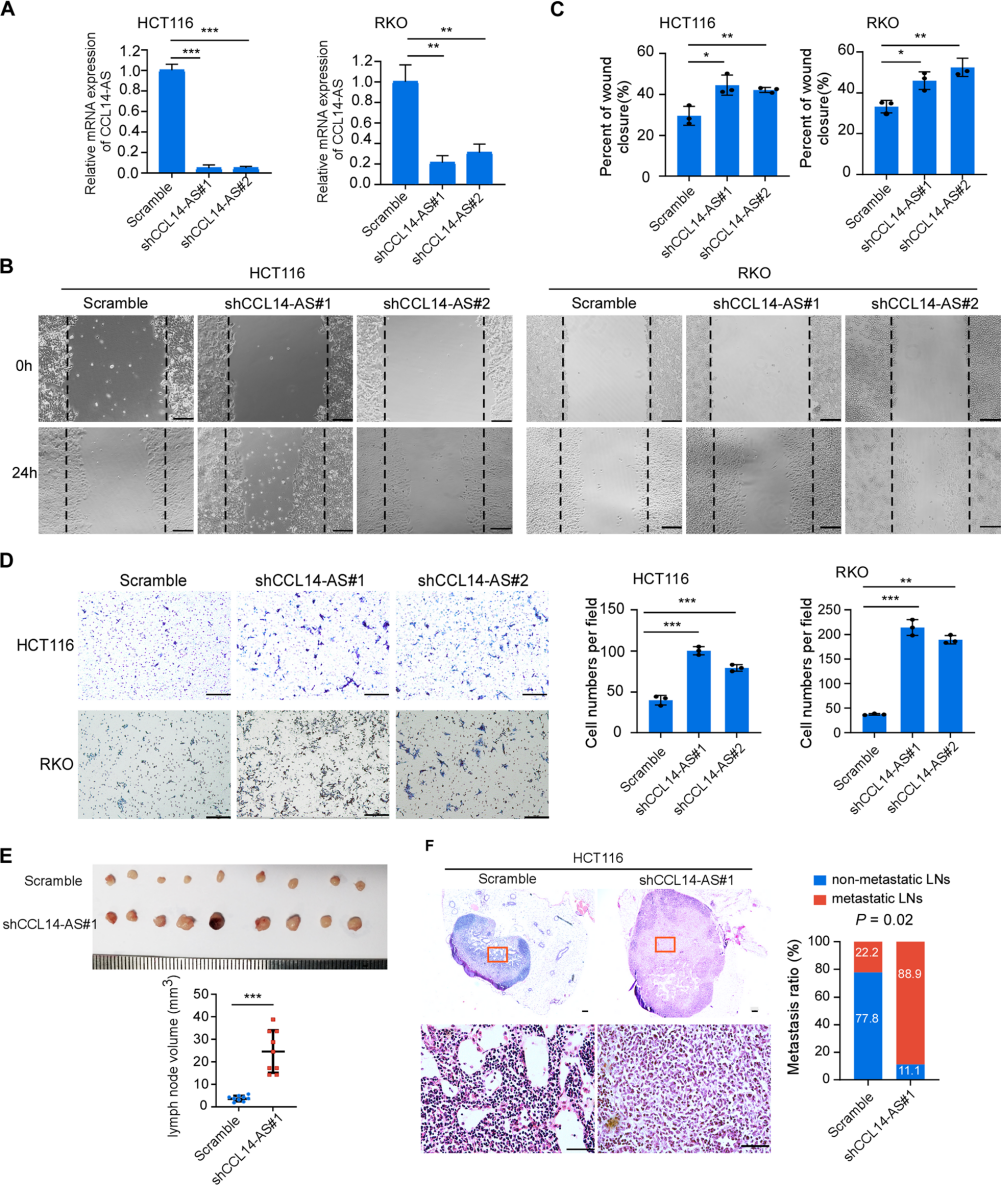
Knockdown of CCL14-AS promotes invasiveness and lymph node metastasis of CRC cells.** A** Detection of CCL14-AS level by qRT-PCR in CCL14-AS-konckdowned HCT116 and RKO cells. **B**, **C** Wound healing assay showing cell migration of HCT116 and RKO cells with or without CCL14-AS knockdown. Scale bar: 100 μm. **D** Migration and invasion of HCT116 and RKO cells with or without CCL14-AS knockdown were evaluated by using transwell assays. Scale bar: 500 μm. **E** Popliteal lymph nodes volume and size were significantly increased in the CCL14-AS knockdown group compared with the control group. **F** Representative images of the lymph node stained with H&E (left) in the CCL14-AS knockdown group. Scale bar: 100 μm. Statistical results of the metastatic ratios of lymph nodes in the Scramble control and CCL14-AS knockdown group (right). * *p* < 0.05, ** p < 0.01, *** p < 0.001

Next, we validated the role of CCL14-AS in invasiveness and LN metastasis by knocking down endogenous CCL14-AS in HCT116 and RKO CRC cell lines, which showed relatively high expression of CCL14-AS (Fig. 3A). MTT assays and colony formation assays showed knockdown of CCL14-AS has no effects on the proliferation of both RKO and HCT116 cells (Additional file 2: Figure S2C-S2D). However, wound healing assays and migration assays showed that knockdown of CCL14-AS dramatically increased the migration and invasion of CRC cells (Fig. 3B–D). Furthermore, knockdown of CCL14-AS significantly promoted the LN metastasis in nude mice (Fig. 3E, F). Taken together, these data showed that lncRNA CCL14-AS suppresses invasiveness and LN metastasis of CRC.

### CCL14-AS interacts with MEP1A mRNA and reduces its stability

**Fig. 4:**
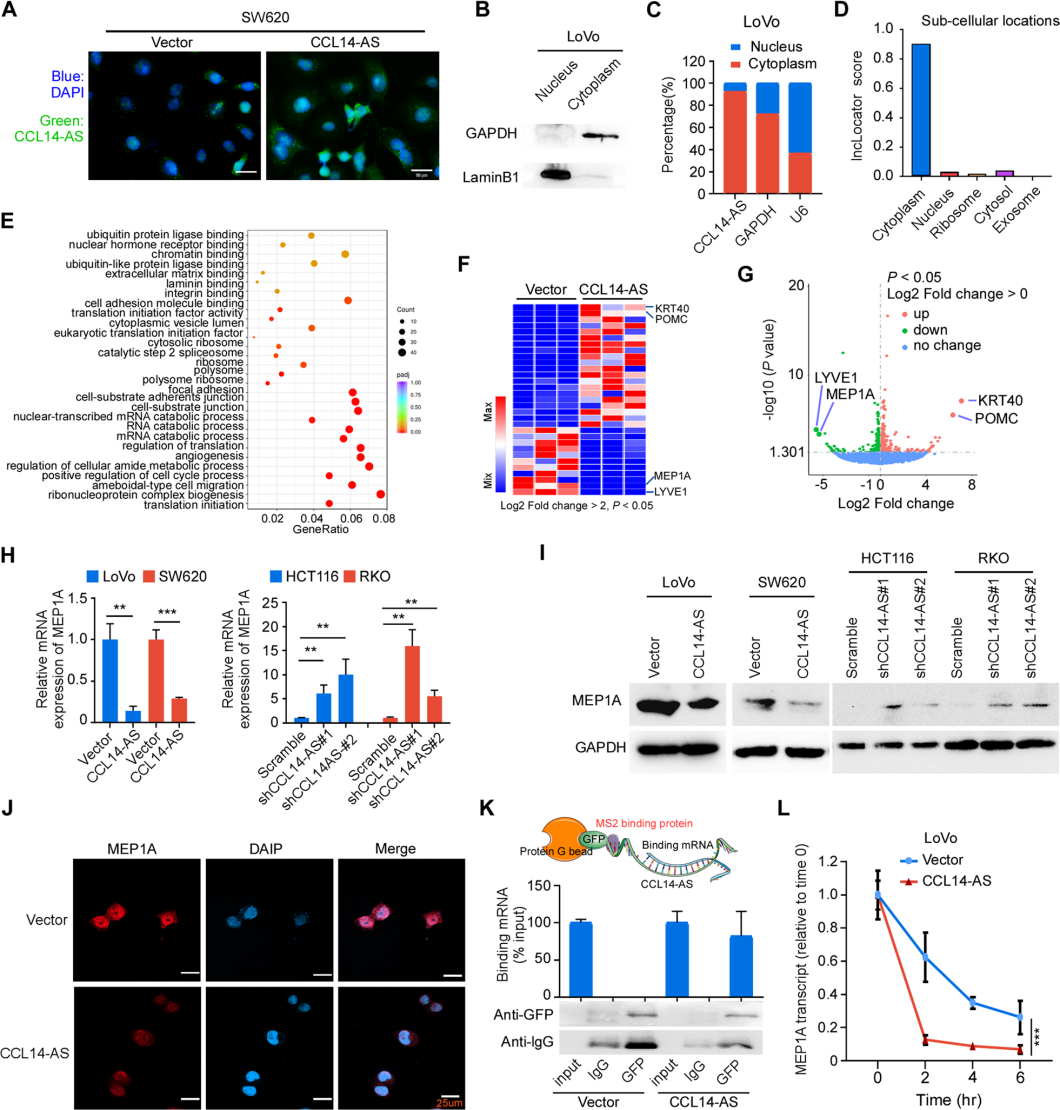
CCL14-AS interacts with MEP1A mRNA and reduces its stability.** A** FISH assay was conducted to determine the subcellular location of CCL14-AS in SW620 cells. DAPI‑stained nuclei are blue. Scale bar: 20 μm. **B–D** RNA fraction assay and online software LncLocator confirmed the subcellular location of CCL14-AS. **B** Western blot analysis detection of the efficiency of nuclear and cytosolic fractionation. **C** Nuclear fractionation analyses and qRT-PCR analyses of CCL14-AS expression in the nucleus and cytoplasm. **D** LncLocator predicts the localization of CCL14-AS in cell. **E** Gene ontology (GO) analyses of differentially expressed genes from RNA-seq assay of stable CRC cells expressing vector or CCL14-AS. **F**–**G** Heatmap **(F)** and volcanic map **(G)** showed significant differentially expressed genes after RNA-seq screening. **H** qRT-PCR confirmed that after overexpressing of CCL14-AS, the mRNA levels of MEP1A decreased, while knockdown of CCL4-AS increased the MEP1A expression. **I**–**J** Western blot analysis **I** and immunofluorescence assay **(J)** both confirmed that overexpressing CCL14-AS reduced MEP1A protein level. Scale bar: 25 μm. **K** MS2-RIP experiments detect the direct binding of CCL14-AS to MEP1A mRNA in CRC cells. **L** RNA-stability analysis showed that CCL14-AS promote the degradation of MEP1A mRNA. ** p < 0.01, *** *p* < 0.001

Next, we sought to unveil the mechanism by which CCL14-AS suppressed invasiveness and LN metastasis. The subcellular location of CCL14-AS in CRC cells was examined by FISH, showing that CCL14-AS was mainly localized in the cytoplasm of SW620 cells (Fig. 4A). Overexpression of CCL14-AS in SW620 cells further increased the amount of cytoplasm enriched CCL14-AS (Fig. 4A). Nuclear and cytoplasmic RNA fractions were prepared from LoVo cells to confirm the subcellular localization of CCL14-AS. The results also demonstrated that CCL14-AS was mainly enriched in the cytoplasm (Fig. 4B, C). These findings were further confirmed by online software LncLocator analyses (Fig. 4D), indicating that CCL14-AS is a cytoplasic-enriched lncRNA in CRC cells.

LncRNAs located in the cytoplasm can mediate signal transduction pathways and translational programs by posttranscriptional control of gene expression [29]. For examples, cytoplasmic lncRNAs can modulate protein posttranslational modifications [30], or regulate mRNA translation and stability [31, 32]. To explore the potential downstream target genes of CCL14-AS, RNA-seq was performed. Differentially expressed genes (DEGs) analysis revealed a series of genes with dramatic difference in their expression. GO analysis showed that the DEGs were significantly enriched in RNA catabolic processes, cell-substrate adherent junction, extracellular matrix binding, regulation of translation initiation, ameboidal-type cell migration, and angiogenesis (Fig. 4E). Among these DEGs genes, LYVE1 (Lymphatic Vessel Endothelial Hyaluronan Receptor 1) and MEP1A (Meprin A Subunit Alpha) were the top 2 downregulated genes, while KRT40 (Keratin 40) and POMC (Pro-opiomelanocortin) were the top 2 upregulated genes upon CCL14-AS overexpression (Fig. 4F, G). Software IntaRNA [33] was applied to predict whether these 4 genes could potentially interact with CCL14-AS. This in silico analysis revealed that CCL14-AS was predicted to interact with MEP1A mRNA and LYVE1 mRNA (Additional file 3: Fig S3). Since the LYVE1 is mainly expressed in endothelial cells and immune cells (THE HUMAN PROTEIN ATLAS), we focus on MEP1A for further investigation in the current study. Consistent with the RNA-seq results, qRT-PCR displayed that overexpression of CCL14-AS significantly decreased, while knockdown of CCL14-AS increased the expression of MEP1A, at both mRNA and protein levels (Fig. 4H–J).

To further elucidate the mechanisms regulating MEP1A expression by CCL14-AS in CRC, we performed RNA immunoprecipitation (RIP) experiments. MS2-RIP experiments revealed that CCL14-AS interacted with MEP1A mRNA (Fig. 4K). RNA-stability analysis showed that CCL14-AS significantly promoted the degradation of MEP1A mRNA (Fig. 4L). Altogether, these data suggested MEP1A is a target mRNA of CCL14-AS in the cytoplasm of CRC cells.

### CCL14-AS suppresses CRC migration and LN metastasis by regulating MEP1A

**Fig. 5:**
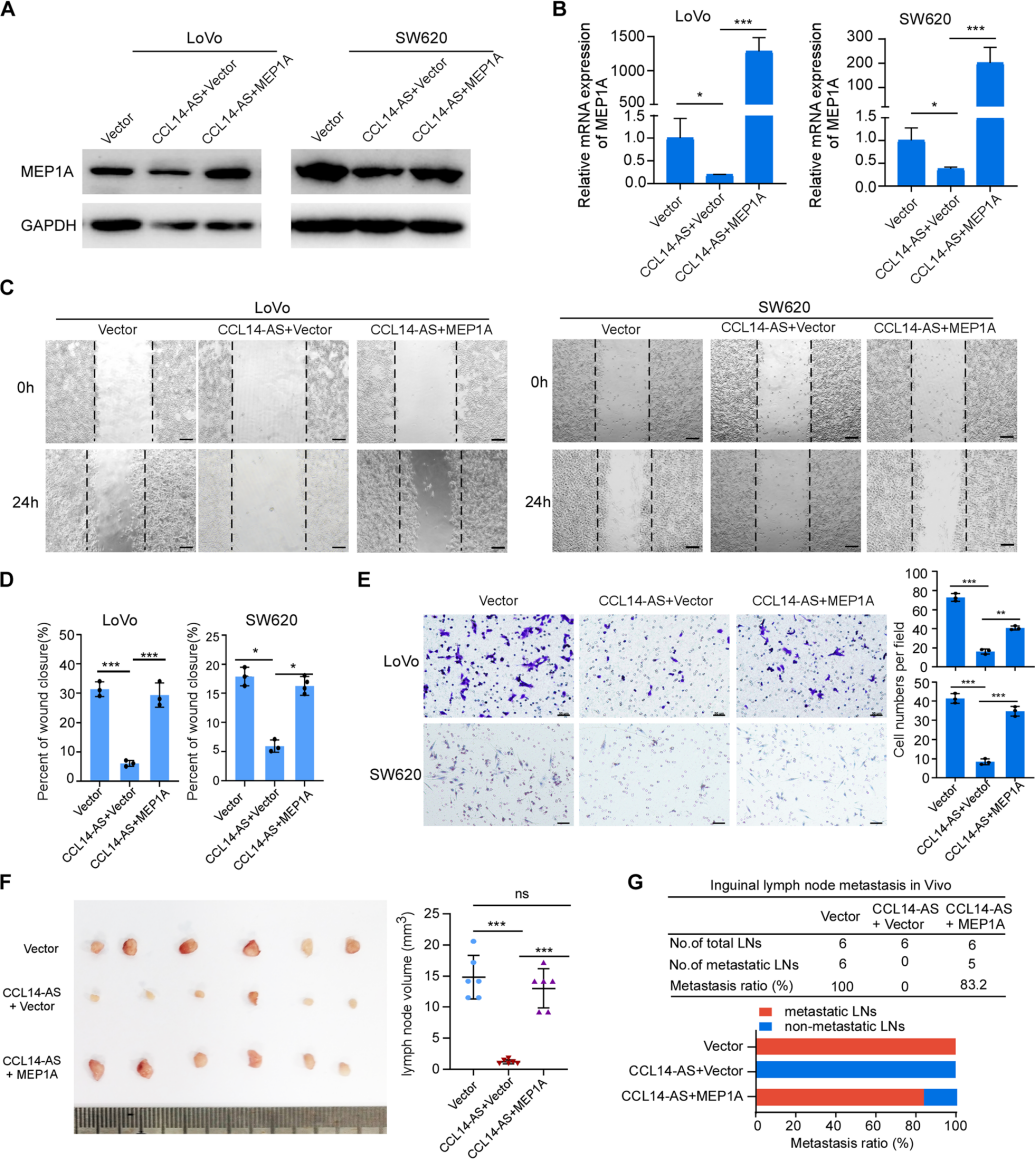
CCL14-AS Inhibits CRC invasiveness by down-regulating MEP1A.** A**, **B** Western blot **(A)** and qRT-PCR **(B)**analyses were performed to examine the expression levels of MEP1A in CCL14-AS overexpressed LoVo and SW620 cells after MEP1A ectopic overexpression. **C–E** Wound healing assay **(C-D)** and Migration assays **(E)** showed that MEP1A ectopic overexpression could prevent the effect of CCL14-AS in inhibiting tumor invasion ability. Scale bar: 100 μm or 50 μm. **F**, **G** Popliteal lymph node metastasis model determined MEP1A could reduce the repressive effects of CRC LN metastasis by CCL14-AS. ns: no significance, * *p* < 0.05, ** *p* < 0.01, *** p < 0.001

It has been reported that MEP1A is upregulated in CRC. Overexpression of MEP1A promotes migration and metastasis ability of CRC cells [34]. We hypothesized that MEP1A might be a key CCL14-AS downstream regulator that promotes progression of CRC. To confirm this hypothesis, MEP1A were overexpressed in CCL14-AS overexpressing cells (Fig. 5A-B). Wound-healing assays and migration assays showed that the ectopic expression of MEP1A significantly reduced the repressive effects on migration and invasion of CRC cells mediated by CCL14-AS in vitro (Fig. 5C–E). Popliteal lymph node metastasis model demonstrated that ectopic expression of MEP1A significantly reduces the repressive effects of lymph node metastasis mediated by CCL14-AS (Fig. 5F, G).

### MEP1A is up-regulated in CRC and negative correlates with CCL14-AS

**Fig. 6:**
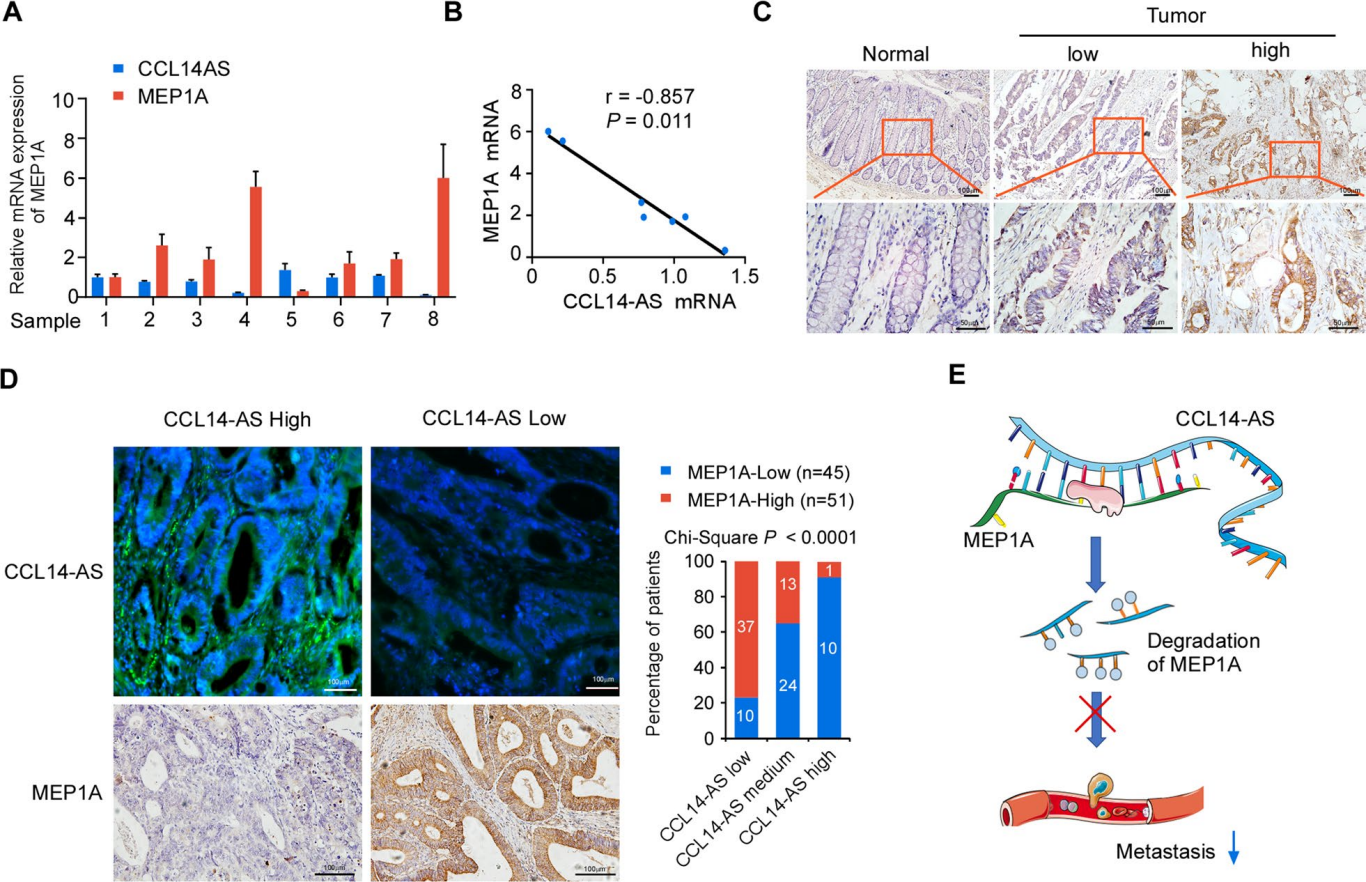
CCL14-AS expression negatively correlates with MEP1A expression in CRC.** A** qRT-PCR analysis was performed to examine the CCL14-AS and MEP1A mRNA expression in eight human CRC tissues. **B** Correlation analysis of CCL14-AS and MEP1A at the mRNA (r = 0.857,* p* = 0.011) in 8 fresh CRC tissues. **C** The expression of MEP1A in normal intestinal tissues and CRC tissues was examined by IHC. Scale bar: 50 μm. **D** The expressions of CCL14-AS and MEP1A protein in specimens, including 96 CRC tissue specimens, were examined by FISH and IHC respectively. Scale bar: 100 μm. Representative FISH or IHC images (left) and correlation analysis (right) of CCL14-AS and MEP1A expression. **E **A working model of CCL14-AS biological function

To assess a potential link between CCL14-AS and MEP1A expression in human CRC, we analyzed the expression levels of CCL14-AS and MEP1A in 8 pairs of matched CRC samples and adjacent noncancerous tissues. The results showed that both the mRNA level and protein level of MEP1A are significantly higher in CRC tissues than in the adjacent tissues (Fig. 6A). Statistical analyses showed that high MEP1A mRNA expression was negatively correlated with CCL14-AS expression in these samples (Fig. 6B). By analyzing the MEP1A protein expression levels using IHC, we found that MEP1A was significantly elevated in CRC tissues as compared in adjacent non-cancerous tissues, which is in consistent with a previous study [34] (Fig. 6C). Moreover, the analysis of CRC tissue microarray containing 96 cases of CRC tissues revealed the significant negative correlation between CCL4-AS expression and MEP1A expression (Fig. 6D).

## Discussion

Numerous studies have revealed that upregulation or downregulation of lncRNA expression has been found in CRC patients. These dysregulated lncRNAs may serve as oncogenes or tumor suppressors. For example, lnc-GNAT1-1 was downregulated in CRC. Low lnc-GNAT1-1 expression was associated with unfavorable clinicopathological features and a poor survival of CRC patients. Knockdown of lnc-GNAT1-1 inhibited the aggressive phenotypes of CRC cell lines and their ability of metastasis to the liver [35]. LINC01082 was downregulated in CRC tissues. Ectopic expression of LINC01082 suppressed the proliferation, migration, and invasion of CRC cells [36]. On the contrary, there are other lncRNAs function as oncogenes in CRC. For example, CCAT1 and CCAT2 expression were upregulated in CRC. CCAT1 alone or in combination with CCAT2 associates with poor survival in CRC patients, and serves as an important prognostic biomarker in CRC [37]. NEAT1 expression is upregulated in CRC and correlates with advanced disease stages and poor overall survival. Overexpression of NEAT1 promotes cell viability and metastasis ability of CRC cells [38]. Studies focusing on the precise molecular mechanisms by which lncRNAs function in LN metastatic CRC are still limited.

In the current study, we identified CCL14-AS,a novel lncRNA, that is associated with disease progression and metastasis in CRC.CCL14-AS expression is downregulated in CRC tissues as compared with adjacent normal intestine tissues. Low CCL14-AS expression was associated with advanced T classification, LN metastasis, distant metastasis, and shorter disease-free survival of CRC patients. Such novel data suggest that the levels of CCL14-AS may be valuable for prognosis of CRC if validated. In addition, CCL14-AS overexpression significantly weakened the invasiveness and LN metastasis ability of CRC cells. These data supported that CCL14-AS may serve as a potential tumor suppressor and a biomarker for LN metastasis in CRC. Moreover, CCL14-AS downregulated MEP1A expression through decreasing its mRNA stability. Furthermore, overexpression MEP1A rescued the CCL14-AS mediated suppression of invasiveness and LN metastasis of CRC cells. Accordingly, CCL14-AS may be a new therapeutic target and interventional strategy to descrease MEP1A may be valuable for control of CRC.

As a novel lncRNA, the functional role and underlying mechanisms of CCL14-AS are unclear. To address this question, RNA-seq was performed to identify the pathways that may affected by CCL14-AS in CRC cells. Interestingly, the DEGs by CCL14-AS overexpression were significantly enriched in RNA catabolic processes, cell-substrate adherent junction, extracellular matrix binding, regulation of translation initiation, ameboidal-type cell migration, and angiogenesis. These results strongly suggested a role of CCL14-AS in invasion and metastasis of tumor cells. Further analysis indicated that LYVE1 and MEP1A might be downstream effectors of CCL14-AS. As a specific marker for lymphatic endothelial cells [42, 43], LYVE1 could be an interesting candidate of CCL14-AS downstream target that mediate invasion and LN metastasis. In addition, it has been reported that high LYVE-1-positive lymphatic vessel numbers were associated with poor prognosis in breast cancer [44]. Since LYVE1 was mainly localized in the endothelial cells while not in epithelial cells, we considered that it might not be a major functional effector downstream of CCL14-AS in CRC cells. However, the regulation of LYVE1 by CCL14-AS could exist in other types of cells. MEP1A is a membrane-bound and secreted astacin metalloproteinases belongs to the metzincin superfamily [45]. Abnormal MEP1A expression has been implicated in several cancers, including CRC [34, 46–48], breast cancer [49], and hepatocellular carcinoma [50]. As a protease that is known to cleave extracellular matrix components in vitro, MEP1A may contribute to tumor progression by facilitating migration, invasion, and metastasis of tumor cells. In CRC, MEP1A activated the EGFR signaling by promoting the shedding of the ligand, thereby activating the ERK/ZEB1 pathway to promote proliferation and migration of CRC cells [48]. These studies suggested that MEP1A is an important regulator that involves in CRC invasion and metastasis. However, the mechanisms that mediated the high expression level of MEP1A in CRC is unclear. In the current study, we showed that CCL14-AS interacted with MEP1A mRNA and promoted its degradation, leading to downregulation of MEP1A. The silence or downregulation of CCL14-AS increased the stability of MEP1A mRNA, resulting in upregulation of MEP1A in CRC. Fascinatedly, CCL14-AS have no effect on CRC cells proliferation. This result may show that CCL14-AS/MEP1A is not the only way to regulate proliferation of CRC cells. There may be the regulatory network to regulate proliferation of CRC cells.

The subcellular localization of lncRNA is closely related to its biological function [29]. Cytoplasm-enriched lncRNAs are widely involved in mediating signal transduction pathways and translational programs [51]. They have essential roles in regulating mRNA translation [31, 32, 52] and in modulating protein posttranslational modifications [30, 53]. They may also serve as competing endogenous RNAs [54] or precursors of microRNAs [55]. There are a few cytoplasm-enriched lncRNAs that target mRNA transcripts and modulate their stability. For example, lncRNA BACE1-AS increases BACE1 mRNA stability in Alzheimer's disease [56]. Overexpression of 1/2-sbsRNAs promotes the degradation of the target mRNAs, while depletion of 1/2-sbsRNAs led to the stabilization and upregulation of the target mRNAs [32, 57]. In the present study, we demonstrated that CCL14-AS was enriched in the cytoplasm of CRC cells. MS2-RIP assays revealed a direct interaction between CCL14-AS and MEP1A mRNA. Overexpression of CCL14-AS lead to increased degradation and downregulation of MEP1A. Interestingly, found that double‑stranded RNA‑binding protein Staufen homolog 1 (STAU1) is one of CCL14-AS binding proteins were predicted by catRAPID. STAU1-mediated mRNA decay (SMD) is a common mRNA degradation process in mammalian cells. 1/2-sbsRNAs mediate mRNA decay by recruiting STAU1 to target mRNAs [32]. Thus, we speculated that CCL14-AS may promote MEP1A mRNA decay through recruiting STAU1 to MEP1A mRNA.

## Conclusion

In summary, we identify a novel lncRNA, CCL14-AS, involved in the progression of CRC. CCL14-AS is downregulated in CRC and inhibits the invasiveness and LN metastasis in CRC. We also provide evidence that the metalloprotease MEP1A may represent a downstream effector of CCL14-AS in the cancer cells. CCL14-AS is mainly localized in the cytoplasm of CRC cells and interacts with MEP1A mRNA to promote the degradation of MEP1A mRNA. Our results suggested that CCL14-AS may be a potential diagnostic biomarker and therapeutic target for CRC (Fig. 6E).

## Supplementary Information

**Additional file 1**: **Figure S1. **Bioinformatics analysis of the fundamental properties of CCL14-AS. **A **Model diagram of CCL14-AS in the genome. **B **Online software the Coding Potential Assessment Tool (CPAT) predict the Coding ability of CCL14-AS. Blue is noncoding RNA, while red is the coding gene. **C **The predicted secondary structure (left) and mountain plot (right) representing the MFE (Red), thermodynamic ensemble of RNA (Green), and centroid (Blue) structures of CCL14-AS.

**Additional file 2**: **Figure S2. **CCL14-AS had no effect on proliferation of CRC cells. **A–B **The LoVo and SW620 stable cells overexpressing CCL14-AS were subjected to MTT assays **A **and Colony-formation assays **B**. in. **C–D **The HCT116 and RKO stable cells expressing shRNA against CCL14-AS were subjected to MTT assays **C **and Colony-formation assays **D**. in. ns: no significance, * *p *< 0.05.

**Additional file 3**: **Figure S3. **LncRRIsearch database predicts the binding capacity of CCL14-AS to MEP1A or LYVE1 mRNAs. A-B Prediction of binding capacity of CCL14-AS to MEPA1 **A **or LYVE1 **B **by LncRRIsearch database.

**Additional file 4**: **Table S1**. List of cancer-related lncRNAs (lncRNAs differently expressed in CRC tissues compared with).

**Additional file 5**: **Table S2**. List of metastasis-related lncRNAs (differently expressed lncRNA in metastatic samples compared with non-metastatic samples（log2FC<-0.5, adj.P.val<0.001)).

**Additional file 6**: **Table S3**. List of survival-related lncRNAs.

**Additional file 7**: **Table S4**. The coding potential of CCL14-AS predicted by the National Center for Biotechnology.

## Data Availability

The datasets generated or analyzed for this study will be made available by the corresponding author on reasonable request.

## Abbreviations

CRC: Colorectal cancer
CCL14: C–C motif chemokine ligand 14
CPAT: Coding potential assessment tool
H&E: Haematoxylin and eosin
lncRNAs: Long non-coding RNAs
IHC: Immunohistochemical
ISH: In situ hybridization
KRT40: Keratin 40
LN: Lymph node
LYVE1: Lymphatic vessel endothelial hyaluronan receptor 1
MEP1A: Meprin A subunit alpha
NCBI: The national center for biotechnology information
OS: Overall survival rate
PRC2: Polycomb repressive complex 2
PFA: Paraformaldehyde
POMC: Pro-opiomelanocortin
RIP: RNA immunoprecipitation
shRNA: Short hairpin RNA
STAU1: Staufen homolog 1
SMD: STAU1-mediated mRNA decay
TCGA: The cancer genome atlas
