# Correction: Long non-coding RNA CCL14-AS suppresses invasiveness and lymph node metastasis of colorectal cancer cells by regulating MEP1A

**DOI:** 10.1186/s12935-024-03582-0

**Published:** 2024-12-28

**Authors:** Mingzhou Li, Chengmei Huang, Yuanyuan Wu, Lina Zhu, Yaxin Zhang, Yi Zhou, Huali Li, Zhihao Liu, Xinyan Pan, Xin Wang, Junfeng Qiu, Fengtian Li, Wenting Liao

**Affiliations:** 1 State Key Laboratory of Oncology in South China, Collaborative Innovation Center for Cancer Medicine, Sun Yat-sen University Cancer Center, Guangzhou, China; 2 Department of Pathology, Nanfang Hospital and School of Basic Medical Sciences, Southern Medical University, Guangzhou, 510515 China; 3 Department of Biomedical Sciences, City University of Hong Kong, Hong Kong, China

**Correction: Cancer Cell International (2023) 23:27**

10.1186/s12935-023-02866-1.

In this article [1], Fig. 5C right panel (SW620 panel): The image of wound healing assay in Vector (0 h) was duplicated with the image in CCL14-AS + Vector (24 h).

The incorrect and correct Fig. 5 are given below:

Incorrect Fig. 5:

**Figure:**
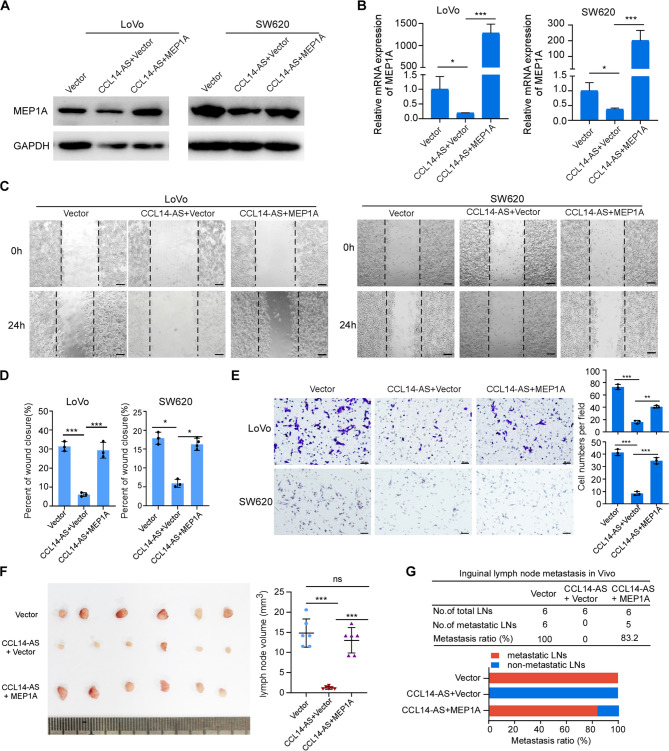

Correct Fig. 5:

**Figure:**
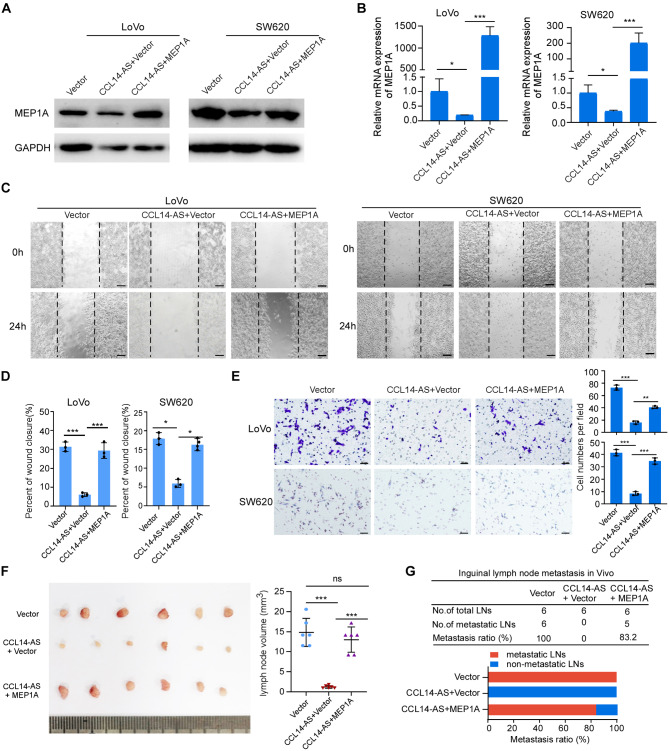

## References

[1] Li M, Huang C, Wu Y, Zhu L, Zhang Y, Zhou Y, Li H, Liu Z, Pan X, Wang X, Qiu J. Long non-coding RNA CCL14-AS suppresses invasiveness and lymph node metastasis of colorectal cancer cells by regulating MEP1A. Cancer Cell Int. 2023;23(1):27. 36793075 10.1186/s12935-023-02866-1 PMC9933342

