# Supplementary figures and images for: Long non-coding RNA CCL14-AS suppresses invasiveness and lymph node metastasis of colorectal cancer cells by regulating MEP1A
Source: Cancer Cell Int. 2023 Feb 15;23:27. doi: 10.1186/s12935-023-02866-1 (PMC9933342; doi:10.1186/s12935-023-02866-1)

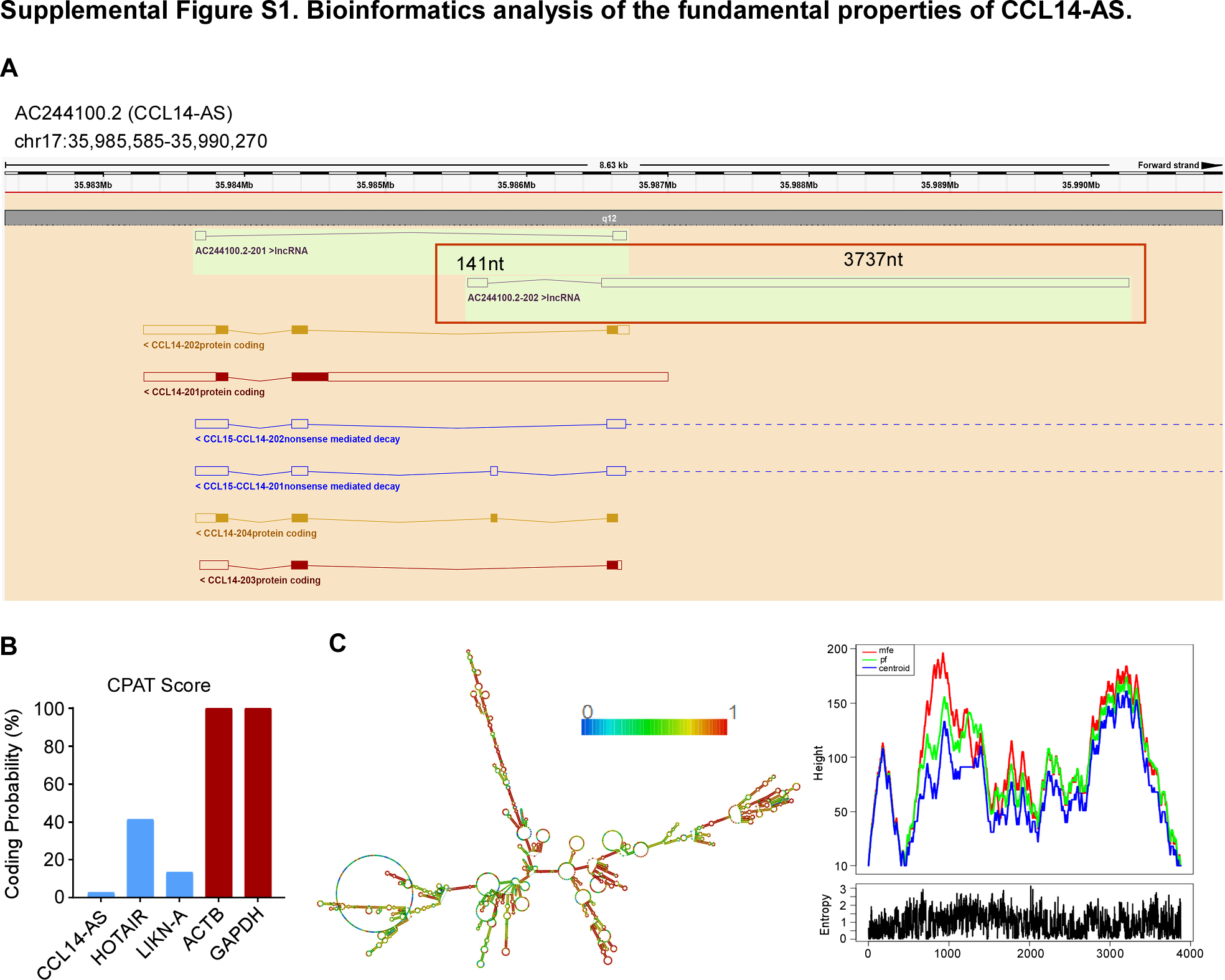

Supplement: Supplementary file 1 — Additional file 1: Figure S1. Bioinformatics analysis of the fundamental properties of CCL14-AS. A Model diagram of CCL14-AS in the genome. B Online software the Coding Potential Assessment Tool (CPAT) predict the Coding ability of CCL14-AS. Blue is noncoding RNA, while red is the coding gene. C The predicted secondary structure (left) and mountain plot (right) representing the MFE (Red), thermodynamic ensemble of RNA (Green), and centroid (Blue) structures of CCL14-AS. [file 12935_2023_2866_MOESM1_ESM.tif]

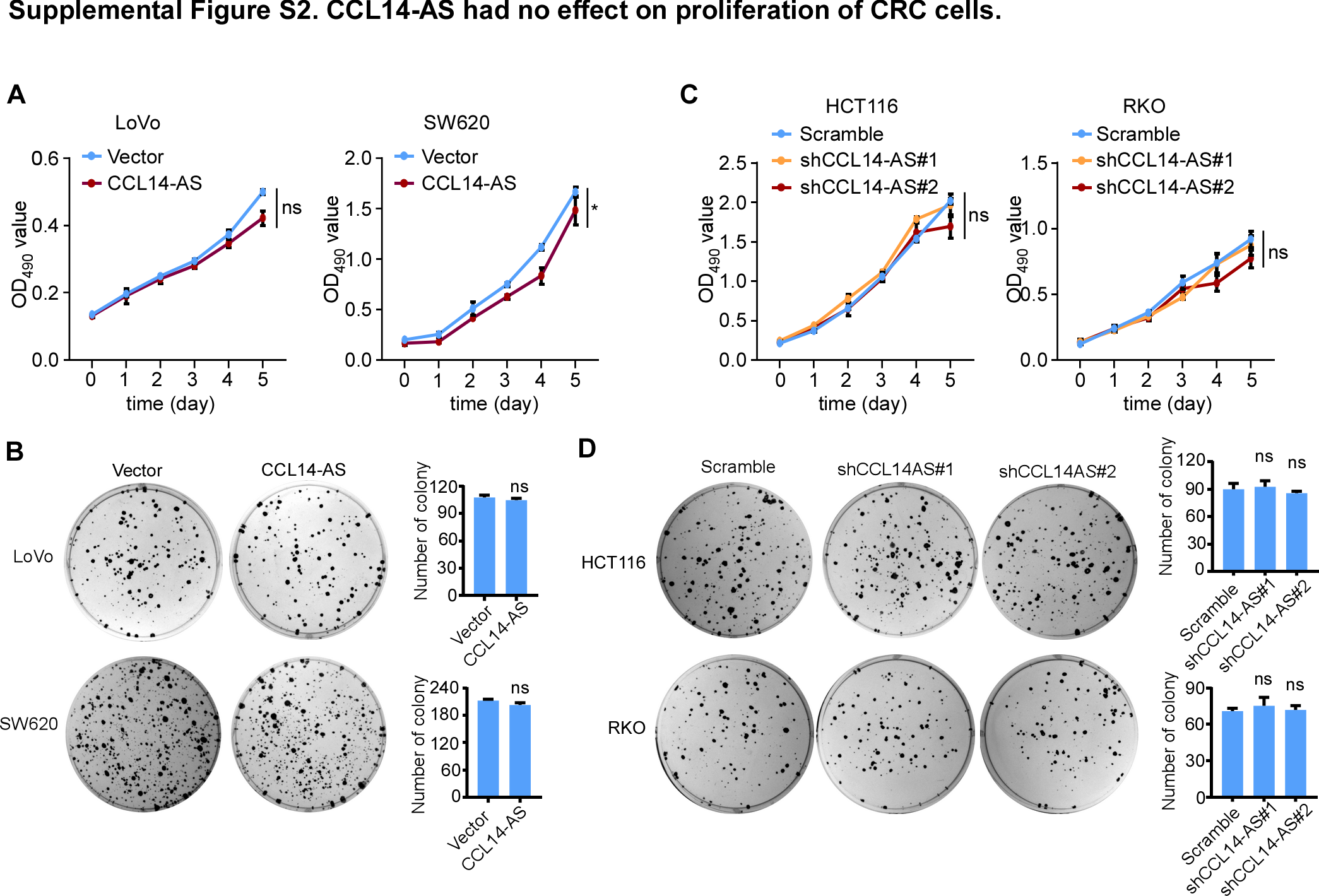

Supplement: Supplementary file 2 — Additional file 2: Figure S2. CCL14-AS had no effect on proliferation of CRC cells. A–B The LoVo and SW620 stable cells overexpressing CCL14-AS were subjected to MTT assays A and Colony-formation assays B. in. C–D The HCT116 and RKO stable cells expressing shRNA against CCL14-AS were subjected to MTT assays C and Colony-formation assays D. in. ns: no significance, * p < 0.05. [file 12935_2023_2866_MOESM2_ESM.tif]

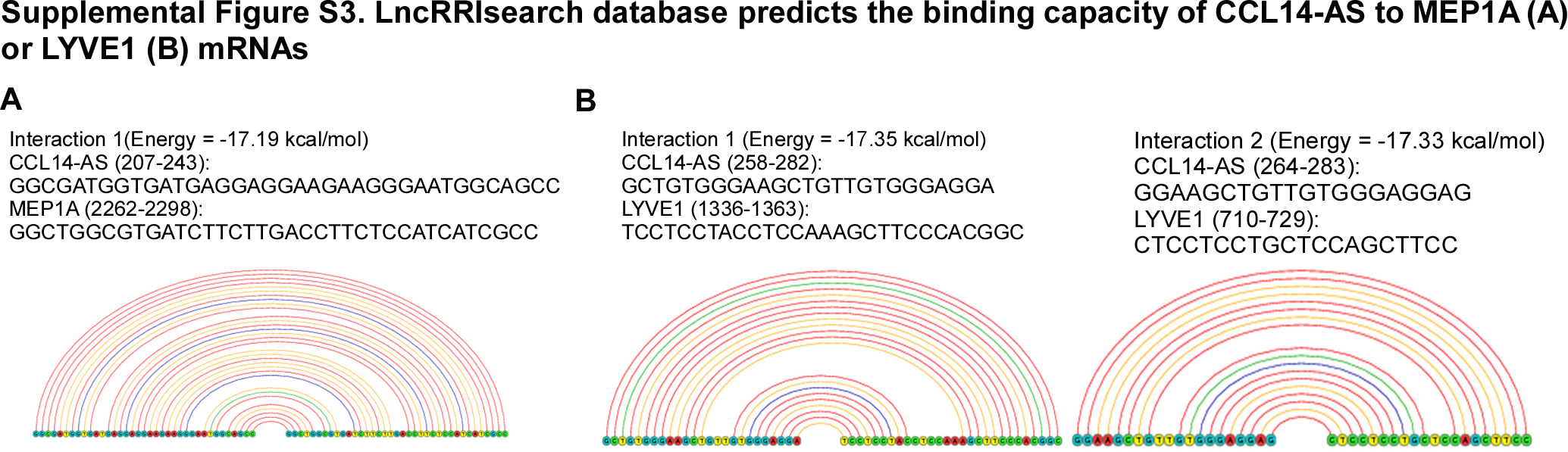

Supplement: Supplementary file 3 — Additional file 3: Figure S3. LncRRIsearch database predicts the binding capacity of CCL14-AS to MEP1A or LYVE1 mRNAs. A-B Prediction of binding capacity of CCL14-AS to MEPA1 A or LYVE1 B by LncRRIsearch database. [file 12935_2023_2866_MOESM3_ESM.tif]
